# Impaired Meningeal Lymphatic Flow in NMOSD Patients With Acute Attack

**DOI:** 10.3389/fimmu.2021.692051

**Published:** 2021-06-14

**Authors:** Xinxin Wang, Haiyan Tian, Han Liu, Dongxiao Liang, Chi Qin, Qingyong Zhu, Lin Meng, Yu Fu, Shuqin Xu, Yanping Zhai, Xuebing Ding, Xuejing Wang

**Affiliations:** ^1^ Department of Neurology, The First Affiliated Hospital of Zhengzhou University, Zhengzhou, China; ^2^ Institute of Parkinson and Movement Disorder, Zhengzhou University, Zhengzhou, China

**Keywords:** meningeal lymphatic vessels, neuromyelitis optica spectrum disorders, acute relapse, DCE-MRI, superior sagittal sinus

## Abstract

The meningeal lymphatic vessels (mLVs) in central nervous system (CNS) have been validated by rodent and human studies. The mLVs play a vital role in draining soluble molecules and trafficking lymphocytes, antigens and antibodies from CNS into cervical lymph nodes (CLNs). This indicates that mLVs may serve as a link between the CNS and peripheral immune system, perhaps involving in the neuroinflammatory disease. However, the morphology and drainage function of mLVs in patients with neuroinflammatory disease, such as neuromyelitis optica spectrum disorders (NMOSD), remains unexplored. Using the dynamic contrast-enhanced magnetic resonance imaging (DCE-MRI), we found that slower flow through mLVs along superior sagittal sinus in NMOSD patients with acute attack instead of NMOSD patients in chronic phase. The reduced flow in mLVs correlated with the disease severity evaluated by expanded disability status scale (EDSS). The receiver operating characteristic curve (ROC) indicated DCE-MRI might provide objective evidence to predict the acute relapse of NMOSD through evaluating the function of mLVs. Promoting or restoring the function of mLVs might be a new target for the treatment of NMOSD relapse.

## Introduction

The lymphatic system is an extensive drainage network of tissues, vessels, and organs that transports lymph throughout the body. It is becoming increasingly clear that the lymphatic system plays an integral role in immunity by removing inflammatory mediators, directing immune cell trafficking and coordinating immune responses (1–3). The central nervous system (CNS) is considered an immune-privileged organ, mainly due to the absence of conventional lymphatic vasculature (4, 5). However, meningeal lymphatic vessels (mLVs) have been identified and recognized as the CNS lymphatic system in both rodents and humans (6–8). The discovery of mLVs provides a route for drainage of macromolecules and immune cells trafficking from CNS directly into the deep cervical lymph nodes (CLNs) (9, 10).

Recently, increasing evidences show that mLVs might be involved in regulating immune response and be associated with neuroinflammatory disease. Studies suggest that mLVs could deliver the autoantigens from CNS into CLNs and induce immune response in CNS (9, 11). Furthermore, decreasing the lymphatic drainage under neuroinflammatory conditions could diminish acquisition of encephalitogenic properties by antigen-specific T cells and ameliorate clinical symptoms of mice with experimental autoimmune encephalomyelitis (EAE, an animal model of multiple sclerosis) (10).

Neuromyelitis optica spectrum disorders (NMOSD) is a severe neuroinflammatory, demyelinating disease of CNS with repeated recurrence and poor prognosis (12). It is characterized by the serum antibodies that targeted the water channel aquaporin-4 (AQP4–immunoglobulin G [IgG]), which mainly affects the brain, optic nerves and spinal cord (13, 14). However, a subset of NMOSD patients were with negative-AQP4 antibody and positive-myelin oligodendrocyte glycoprotein (MOG) antibody, and those patients have distinct clinical features, fewer relapses, and better recovery than patients with positive-AQP4 antibody (15, 16). As NMOSD patients with positive-AQP4 antibody were considered to be more readily to relapse than patients with negative-AQP4 antibody (100% vs. 81.5%) (17), it is meaningful to investigate the meningeal lymphatic drainage in these patients and explore the potential target to alleviate and prevent relapse.

NMOSD with positive-AQP4 antibody is a demyelinating disease in CNS which pathogenetic AQP4-IgG mediates and complement participates. Previous vitro studies have showed that binding of AQP4-IgG to AQP4 could initiates complement activation, disruption of both water and glutamate homeostasis (18), astrocyte toxicity (19), membrane lesioning (20, 21), promotion of the migration of granulocytic leukocytes and natural killer cell, and increase of the permeability of the endothelial barrier to plasma proteins (19, 22). Thus, clearing the AQP4 antibody from CNS effectively is another way to prevent the acute exacerbation or relapse of NMOSD. As the mLVs might play an important role in draining immune associated cells, antigens, and antibodies from CNS to peripheral immune system (10), one cannot help but wonder whether the drainage dysfunction of the mLVs is associated with the development of NMOSD. However, there is no direct evidence that meningeal lymphatic flow is measured in NMOSD patients through noninvasive method. The only EAE animal model showed that the decreased lymphatic drainage was related to the clinical phenotype of the animal model (10).

In 2017, the visualization of mLVs around superior sagittal sinus (mLVs-SSS) in human and nonhuman primates has been validated noninvasively *in vivo* by high-resolution MRI scans (23). The clearance of contrast agent in putative mLVs were also evaluated by the head high-resolution T2 fluid-attenuation inversion recovery (T2 flair) imaging discontinuously (24). We have recently assessed the mLVs-SSS flow in patients with idiopathic Parkinson’s disease or atypical Parkinsonian disorders using the dynamic contrast-enhanced MRI (DCE-MRI) (25), which may also be useful to measure abnormal mLVs flow in neuroinflammatory diseases. Here we used DCE-MRI to evaluate the mLVs-SSS flow quantitatively in NMOSD patients with acute attack (ANMOSD), NMOSD patients in chronic phase (CNMOSD), and normal control (NC).

## Materials and Methods

### Approval and Patient Informed Consent

This study was authorized by the Institutional Ethics Committees of The First Affiliated Hospital of Zhengzhou University and written informed consent was obtained from all the subjects.

### Participants

Healthy participants were recruited from the Physical Examination Center of the First Affiliated Hospital of Zhengzhou University. All the healthy participants were in good health evaluated by the physical examination, the laboratory and imaging examination, including the blood routine, urine routine, liver and kidney function, blood glucose and lipids, thyroid function tests, MRI and magnetic resonance angiography scans. All healthy participants were confirmed to be free of neurological and psychiatric disorders, as determined by two attending neurologists and a psychiatrist.

All 68 NMOSD patients were collected from Neurology department of the First Affiliated Hospital of Zhengzhou University from March 2019 to September 2020. All patients included in this study had to meet the following inclusion criteria: (1) diagnosis of NMOSD according to the 2015 International Panel diagnostic criteria for NMOSD with AQP4-IgG (26); (2) CSF positive for AQP4-antibodies; (3) age above 18 years at disease onset; (4) without contraindications for MRI examination; (5) without adverse reaction to gadobutrol and normal renal function; (6) written informed consent. Exclusion criteria were as follows; (1) diagnosis of other type of acquired demyelinating syndromes, such as MS or an infectious, metabolic, vascular, or neoplastic CNS diseases; (2) with contraindications for MRI examination; (3) with adverse reaction to gadobutrol or abnormal renal function. All patients were test for CSF AQP4-IgG using cell-based assay. All patients were interviewed and examined by two board-certified neurologists who had experience with autoimmune diseases. Seven patients were excluded from the research because of discomfort during MRI scans or blurred images, and a total of 61 NMOSD patients whose MRI data were finally analyzed in this study, including 32 ANMOSD patients and 29 CNMOSD patients. The Expanded Disability Status Scale (EDSS) was used to quantify the disability of NMOSD patients. The EDSS steps 1.0 to 4.5 refer to NMOSD patients who were able to walk without any aid, while the EDSS steps 5.0 to 9.5 are defined as NMOSD patients who were with the impairment to walking. Thus, the patients in the ANMOSD were divided into I-ANMOSD (EDSS ≤ 4.5) group and II-ANMOSD (EDSS > 4.5) group. As the EDSS scores of CNMOSD patients were mostly less than 5.0, so the CNMOSD patients were not divided into two groups.

### Imaging Procedures

All examinations were performed on a 3-Tesla MRI unit (Skyra, Siemens Healthcare, Erlangen, Germany) with 20-channel head-neck gradient coil for radiofrequency transmission. The suggested dosing (0.1 mmol/kg) of gadobutrol (Gadovist, Bayer Pharma AG, Berlin, Germany) was injected intravenously, using an automatic high-pressure syringe (Spectris MRI Injector System, Medrad, Indianola, PA, USA).

The MRI protocol included the following:
1) DCE-MRI acquisitions:
In order to evaluate the mLVs-SSS flow, DCE-MRI of the mLVs-SSS, including left, right, and lower mLVs-SSS (L-mLVs-SSS, R-mLVs-SSS, and Lo-mLVs-SSS), was performed after the injection of gadobutrol. The standard 2D T1 black-blood sequences were employed in the DCE-MRI acquisitions in each participant, and each series lasted 16.78 s and contained three contiguous slices with 3 mm thickness. The following parameters in this sequence were as follows: coronal 2D acquisition, repetition time/echo time (TR/TE) = 700/11 ms, field of view (FOV) 170 mm, acquisition matrix 154 × 192, voxel size 0.9 × 0.9 × 3.0 mm^3^, acquisition time 6 min 26 s.
For the location of mLVs-SSS, the axis perpendicular to the tangent line of SSS and passing through the posterior edge of the corpus callosum was drawn as the mid-sagittal axis. The coronal plane in line with the mid-sagittal axis was determined as the standard central coronal plane. MRI scans were moved forward or backward from the standard coronal plane by 2.0 mm to acquire three coronal planes.
2) High-resolution MRI sequences:
Thirty minutes after injection of gadobutrol, each individual underwent three high resolution MRI scans consecutively at one time, including 2D T1 black-blood, 3D T1 black-blood, and 3D T2 flair scans. The cross-sectional areas of mLVs-SSS were measured using the three high-resolution MRI scans, and the sequence parameters were as follows:
(1) Limited T1 black-blood scan: coronal 2D acquisition over the SSS, TR/TE = 707/210 ms, FOV 170 mm, acquisition matrix 256 × 256, voxel size 0.7 × 0.7 × 2.0 mm^3^, five contiguous sections with 2.0 mm thickness, acquisition time 6 min 25 s. The location and central coronal plane were the same as that in DCE-MRI, and the coronal MRI scans were moved forward or backward from the standard coronal plane by 2.0 mm to acquire four coronal planes.
(2) Whole-brain T1 black-blood scan: coronal 3D acquisition, Sampling Perfection with Application optimized Contrasts using different flip angle Evolution (SPACE) sequence, TR/TE = 700/20 ms, FOV 230 mm, acquisition matrix 256 × 256, voxel size 0.9 × 0.9 × 0.9 mm^3^, 112 contiguous sections with 0.9 mm thickness, acquisition time 7 min 06 s. The location of central coronal plane was the same as that in DCE-MRI.
(3) Whole-brain T2 flair scan: coronal 3D acquisition, SPACE, TR/TE = 8000/81 ms, FOV 220 mm, acquisition matrix 224 × 320, voxel size 0.7 × 0.7 × 2.0 mm^3^, 30 contiguous sections with 2.0 mm thickness, acquisition time 4 min 02 s. The location of central coronal plane was the same as that in DCE-MRI.
3) Motion correction:
We took a series of measures to reduce movement artifacts during head MRI scans: (1) Using long-term averaging (LOTA) method to reduce motion artifacts generated by the swallowing or cerebral artery pulsation: (2) During the head MRI scan, folded towels were used to fix the head and neck of participants in the head coil: (3) Taking behavioral interventions to reduce the head motion. The participants were informed to stare at a cross logo which was fixed in the middle and upper part of the machine throughout the MRI scan. Those who could not complete the MRI scans, or whose images were rated as blurred after motion correction were excluded from the analysis in our study.

### Imaging Analysis

The MRI images were analyzed by three expert radiologists with 10 years of MRI experience independently, and each of them was blinded to the patients’ information. The mLVs were tubular-shaped structures with a circular cross section, which mainly run alongside the venous dural sinuses and near the venous sinus in the coronal slices, especially in the SSS. Before injection of gadobutrol, the mLVs could not be discerned from SSS and dura blood vessels. However, after injection of gadobutrol, the dura blood vessels and SSS both darkened in T1 black-blood and T2 flair sequences, while Gd would leak out of blood vessels and collect inside lymphatic vessels in the dura matter, and the mLVs surrounded SSS would show up as white round like areas in both sequences. Regions of interest (ROI) representing the mLVs-SSS was outlined manually by the radiologists expertly according to the DCE-MRI images before and after the gadobutrol injection in the central coronal MRI images. The commercial image viewing software (IntelliSpace Portal v.7, Philips Healthcare) was used to view the 23 series of DCE-MRI images, which do benefit for us to compare the mLVs before and after gadobutrol injection, and this would be convenient for us to draw the ROI of L-, R-, and Lo-mLVs-SSS in DCE-MRI images in Siemens Workstation. The DCE-MRI data were analyzed with post-processing software (syngoMMWP VE40A, Siemens AG).

Scanner-generated DICOM images were used to measure the cross-sectional areas of mLVs-SSS on RadiAnt DICOM viewer software (https://www.radiantviewer.com/dicom-viewer-manual/v/5.0.0/). The five central coronal slices in the high-resolution MRI sequences are referenced to measure the cross-sectional areas of the mLVs-SSS. The largest cross-sectional areas, the average cross-sectional areas, and the minimal cross-sectional areas of the mLVs-SSS in each individual were selected for analysis. The largest, average, and minimal cross-sectional areas of three mLVs-SSS (L-, R-, and Lo-mLVs-SSS) in different groups were calculated and compared separately. The obtained DCE-MRI data were interpreted semi-quantitatively (the TTP and AUC) and quantitatively (wash-in rate) using time-intensity curve (TIC). Using the mean curve function in syngoMMWP VE40A, TIC was generated by averaging signal intensity within each ROI at different time points. The TTP values, wash-in rate values, and AUC values of mLVs-SSS (L-, R-, and Lo-mLVs-SSS) in different groups were compared.

### Statistical Analysis

GraphPad Prism 8.0 software (GraphPad Software Inc., San Diego, CA, USA) and PASW Statistics 18.0 (IBM, Armonk, NY) were used for statistical analysis. The observed clinical and demographic continuous data were expressed by means ± standard (SD). The average cross-sectional area was shown by means ± standard error of the mean (SEM), categorical data and discontinuous variables were expressed by medians, frequencies, and percentages. Mann-Whitney test and Chi-squared Test were used to compare demographic factors and clinical characteristics. Spearman correlation was used to test the association between EDSS scales and DCE-MRI parameters (TTP, wash-in rate, AUC) of L-, R-, and Lo-mLVs-SSS in ANMOSD patients, respectively. Meanwhile, the Spearman correlation analysis was also used to evaluate the correlations between EDSS scales and the largest, average, and minimal cross-sectional areas of L-, R-, and Lo-mLVs-SSS in ANMOSD patients, respectively. The Kruskal-Wallis test followed by Dunn’s multiple comparisons test were used to compare the values of DCE-MRI parameters (TTP, wash-in rate, AUC) of L-, R-, and Lo-mLVs-SSS among three groups (NC vs. ANMOSD vs. CNMOSD group, NC vs. I-ANMOSD vs. II-ANMOSD group), respectively. The one-way ANOVA test followed by Newman-Keuls multiple comparisons test were used to compare the largest, average, minimal cross-sectional areas of L-, R-, and Lo-mLVs-SSS among three groups (NC vs. ANMOSD vs. CNMOSD group, NC vs. I-ANMOSD vs. II-ANMOSD group), respectively. Receiver operating characteristic curve (ROC) analysis was used to evaluate the diagnostic accuracy of DCE-MRI parameters of L-, R-, and Lo-mLVs-SSS in distinguishing ANMOSD patients from CNMOSD patients, or distinguishing II-ANMOSD patients from CNMOSD patients separately. Additionally, ROC curve analysis were used to evaluate the diagnostic capacity of combined DCE-MRI parameters of L+ R+ Lo-mLVs-SSS, L+ R-mLVs-SSS, L+ Lo-mLVs-SSS and R+ Lo-mLVs-SSS in distinguishing ANMOSD patients from CNMOSD patients, or distinguishing II-ANMOSD patients from CNMOSD patients. Logistic regression models were used to analyze the effects of clinical factors (sex, age, disease duration, number of attacks, MRI lesion, EDSS scales) on the DCE-MRI parameters in ANMOSD patients. *P* < 0.05 was considered statistically significant.

## Results

### Demographics and Clinical Characteristics

In this research, 41 NC subjects (9 males and 31 females, mean age 36.0 ± 7.9 years), 32 ANMOSD patients (8 males and 24 females, mean age 36.2 ± 9.2 years), and 29 CNMOSD patients (8 males and 21 females, mean age 37.2 ± 7.4 years) were enrolled in this study and completed the MRI scans successfully. The demographics and clinical characteristics of the NC, ANMOSD and CNMOSD patients were shown in **Table 1**. According to the EDSS, the ANMOSD patients were divided into the I-ANMOSD group (EDSS stage ≤ 4.5, n = 17, 5 males and 12 females, mean age 35.5 ± 9.7 years) and II-ANMOSD group (EDSS stage > 4.5, n = 15, 3 males and 12 females, mean age 37.2 ± 7.4 years). The demographics and clinical characteristics of the NC, I-ANMOSD and II-ANMOSD patients were shown in **Table 2**.

**Table 1:** The demographics and clinical characteristic of subjects.

Characteristic	NMOSD patients			Normal controls
	acute attack (n = 32)	chronic phase (n = 29)	*P* Value	(n = 41)
Age at onset, mean (SD), years	36.2 (9.2)	37.2 (7.4)	0.660	36.0 (7.9)
Female, no. (%)	24 (75.0%)	21(72.4%)	>0.999	31 (75.6%)
Duration, median (range), months	4 (1–36)	42 (11–63)	<0.001	NA
Number of attacks, median (range)	1 (1–3)	3 (2–6)	<0.001	NA
MRI lesion				
Spinal cord, no. (%)	12 (37.5%)	10 (34.5%)	>0.999	NA
Optic nerve, no. (%)	11 (34.4%)	11 (37.9%)	0.796	NA
Brain, no. (%)	7 (21.8%)	5 (17.2%)	0.753	NA
others, no. (%)	2 (6.2%)	3 (10.3%)	0.666	NA
EDSS score	4.5 (1.5-9.0)	2.0 (1.0-5.5)	<0.001	NA
SBP before MRI	123.2 ± 13.1	119.5 ± 13.1	0.289	119.1 ± 14.0
DBP before MRI	77.5 ± 10.9	76.7 ± 11.1	0.782	76.9 ± 8.3
SBP after MRI	120.0 ± 13.5	117.5 ± 13.6	0.479	118.4 ± 11.3
DBP after MRI	80.2 ± 12.4	74.1 ± 13.0	0.089	74.3 ± 9.3
HR before MRI	74.1 ± 11.4	71.1 ± 10.1	0.411	72.3 ± 10.5
HR after MRI	75.2 ± 10.7	72.1 ± 10.1	0.225	73.5 ± 11.8

DBP, diastolic blood pressure; EDSS, expanded disability status scale; HR, heart rate; NA, not applicable; NMOSD, neuromyelitis optica spectrum disorders; SBP, systolic blood pressure.

**Table 2:** The demographics and clinical characteristic of I-ANMOSD and II-ANMOSD.

Characteristic	ANMOSD patients		
	Ⅰ-ANMOSD	Ⅱ-ANMOSD	*P* Value
	(n = 17)	(n = 15)	
Age at onset, mean (SD), years	35.5 (9.7)	37.2 (7.4)	0.633
Female, no. (%)	12 (70.6%)	12 (80.0%)	0.691
Duration, median (range), months	4 (1–36)	6 (2–32)	0.560
Number of attacks, median (range)	1 (1–3)	2 (1–3)	0.188
MRI lesion			
Spinal cord, no. (%)	7 (41.2%)	5 (33.3%)	0.737
Optic nerve, no. (%)	5 (29.4%)	6 (40.0%)	0.712
Brain, no. (%)	4 (23.5%)	3 (20.0%)	>0.999
others, no. (%)	1 (5.9%)	1 (6.7%)	>0.999
EDSS score	3.5 (1.5-4.5)	7.0 (5.0-9.0)	<0.001
SBP before MRI	125.1 ± 13.0	121.1 ± 13.1	0.390
DBP before MRI	75.7 ± 10.6	79.5 ± 11.4	0.340
SBP after MRI	121.6 ± 13.1	118.1 ± 14.2	0.480
DBP after MRI	78.2 ± 9.4	82.4 ± 15.0	0.343
HR before MRI	76.1 ± 10.4	71.8 ± 12.4	0.301
HR after MRI	77.1 ± 9.5	73.1 ± 11.8	0.291

DBP, diastolic blood pressure; EDSS, expanded disability status scale; HR, heart rate; I-NMOSD, neuromyelitis optica spectrum disorders (EDSS scale ≤ 4.5); II-NMOSD, neuromyelitis optica spectrum disorders (EDSS scale > 4.5); SBP, systolic blood pressure.

Before and after MRI scan, we monitored the blood pressure (BP) and heart rates (HR) of each participant. As shown in **Table 1**, no significant difference existed among NC, ANMOSD, and CNMOSD groups in systolic BP (SBP), diastolic BP (DBP) and HR both before and after the MRI scans, respectively.

### Quantitative Assessment of the mLVs-SSS Flow

**Figure 1:**
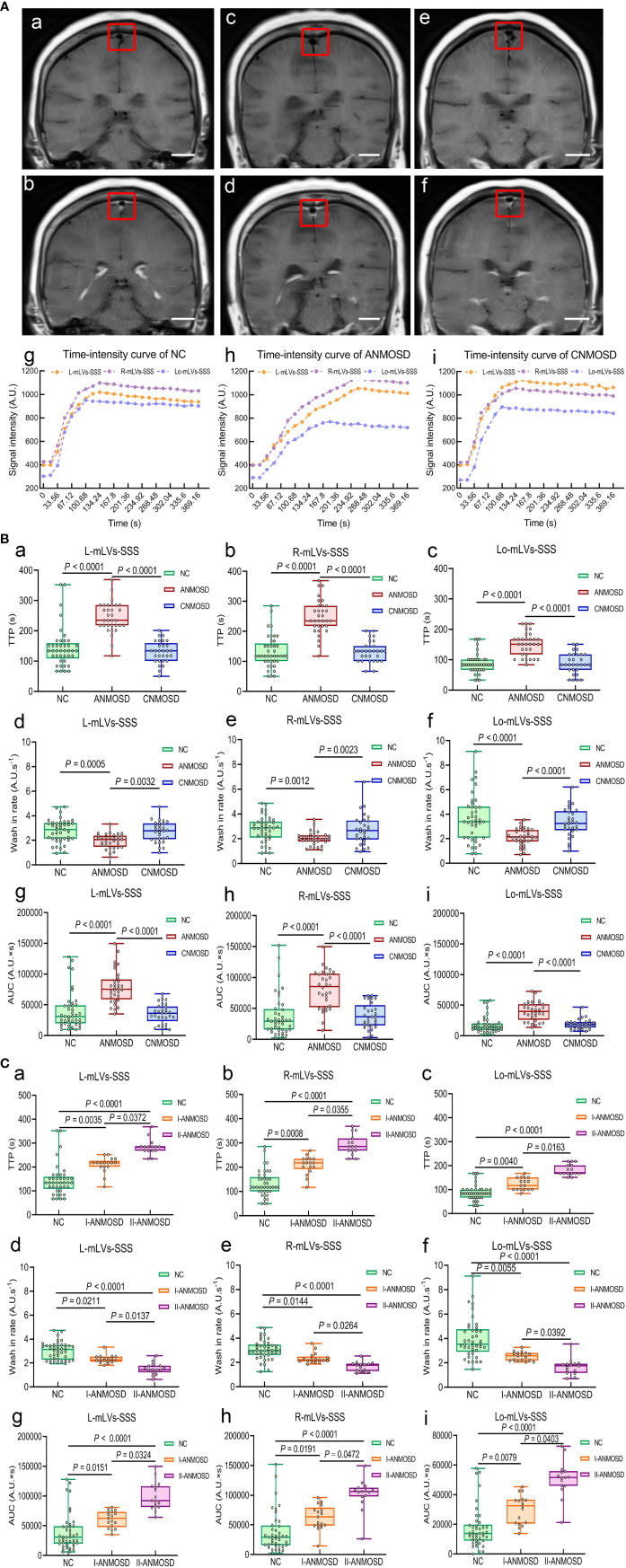
Quantitative assessment of mLVs-SSS flow by DCE-MRI. **(A)** Representative DCE-MRI images of the mLVs-SSS (L-mLVs-SSS, R- mLVs-SSS and Lo- mLVs-SSS) before **(a, c, e)** and after **(b, d, f)** vascular administration of the gadobutrol in NC **(a, b)**, ANMOSD **(c, d)**, and CNMOSD **(e, f)** groups. scale bar, 2 cm. The red rectangles stand for the mLVs-SSS. L-mLVs-SSS, R- mLVs-SSS and Lo- mLVs-SSS represented the left, right and lower mLVs-SSS, respectively. The representative time-intensity curves (TIC) in NC **(g)**, ANMOSD **(h)**, and CNMOSD **(i)** were obtained by DCE-MRI images. **(B)** Comparison of the TTP **(a–c)**, wash-in rate **(d–f)**, and AUC **(g–i)** of mLVs-SSS in NC, ANMOSD, and CNMOSD groups. **(C)** The TTP **(a–c)**, wash-in rate **(d–f)**, and AUC **(g–i)** of mLVs-SSS in NC and ANMOSD patients (I-ANMOSD group, II-ANMOSD group) were further compared. I-ANMOSD group, EDSS scale ≤ 4.5; II-ANMOSD group, EDSS scale > 4.5. TTP, time to peak; AUC, area under curve.

The meningeal lymphatic flow was evaluated by DCE-MRI, which was able to detect the uptake of gadolinium-based contrast media by mLVs-SSS. Representative DCE-MRI images of the mLVs-SSS before (**Figures 1Aa, c, e**) and after (**Figures 1Ab, d, f** **)** vascular administration of the gadobutrol in NC, ANMOSD, and CNMOSD were displayed. Representative pictures of TIC for mLVs-SSS from each group showed that TTP values of mLVs-SSS (L, R and Lo-mLVs-SSS) in ANMOSD group were prolonged (**Figures 1Ag–i** **)**. The statistical results showed that TTP values of mLVs-SSS in ANMOSD patients were significantly prolonged than that in NC and CNMOSD group, however, TTP values of mLVs-SSS in NC and CNMOSD group were not significantly different with each other (**Figures 1Ba–c**). In addition, wash-in rate values of mLVs-SSS in ANMOSD patients were significantly slower than that in NC and CNMOSD group, however, that in NC and CNMOSD patients were not significantly different compared with each other (**Figures 1Bd–f**). Moreover, AUC values of mLVs-SSS in ANMOSD patients were significantly larger than that in NC and CNMOSD patients; however, AUC values of mLVs-SSS in NC and CNMOSD group were not significantly different compared with each other (**Figures 1Bg–i**).

Next, the parameters (TTP, wash-in rate, and AUC) of TIC in I-ANMOSD, II-ANMOSD, and NC groups were further compared. Our findings showed that TTP values of mLVs-SSS (L, R, and Lo-mLVs-SSS) in both I-ANMOSD and II-ANMOSD were significantly prolonged than that in NC, and TTP values of mLVs-SSS in II-ANMOSD were significantly longer than that in I-ANMOSD (**Figures 1Ca–c**). Additionally, wash-in rate values of mLVs-SSS in both I-ANMOSD and II-ANMOSD groups were significantly decreased than that in NC, and wash-in rate values of the mLVs-SSS in I-ANMOSD group were significantly slower than that in II-ANMOSD group (**Figures 1Cd–f**). Moreover, AUC values of mLVs-SSS in I-ANMOSD and II-ANMOSD groups were both significantly larger than that in NC, and AUC values of mLVs-SSS in II-ANMOSD group were significantly increased than those in I-ANMOSD group (**Figures 1Cg–i**).

**Figure 2:**
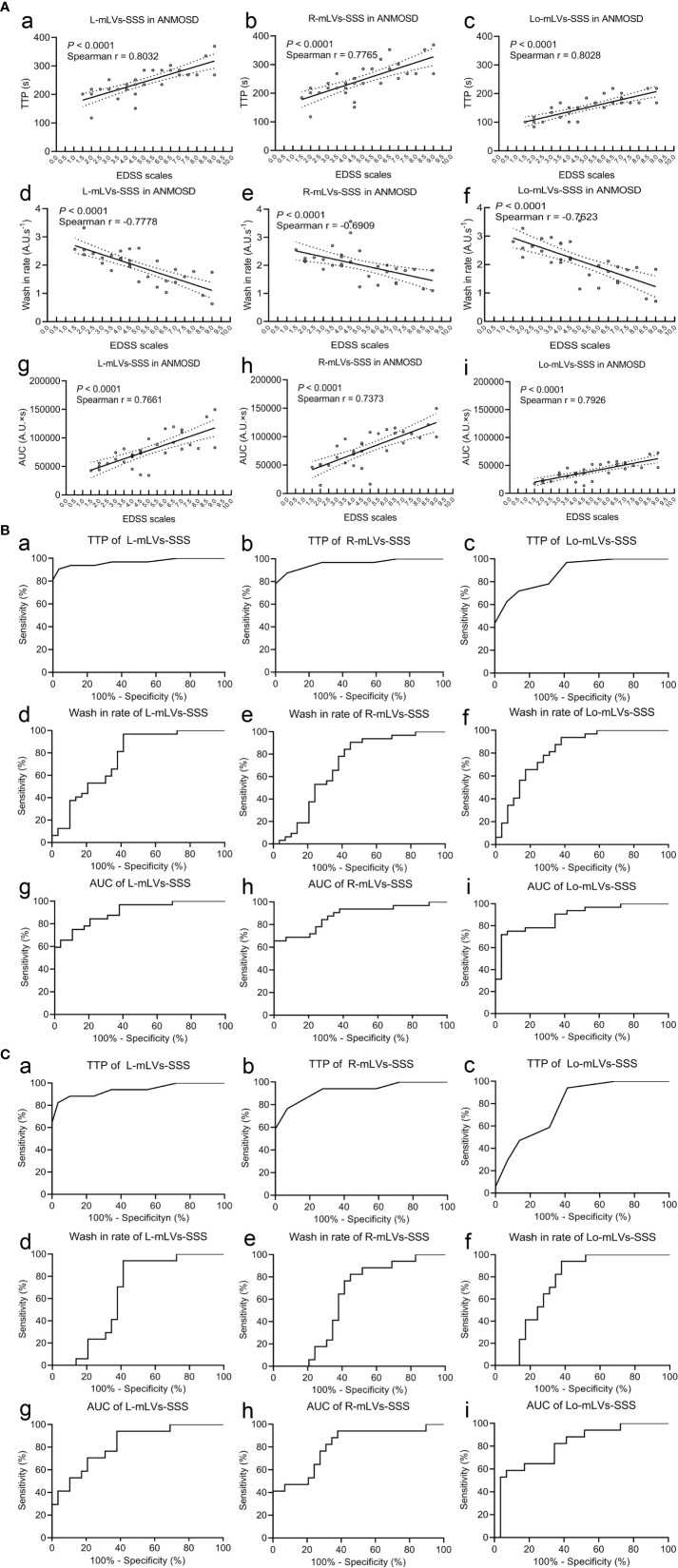
Correlation between EDSS scales and DCE-MRI parameters, and the diagnostic accuracy of DCE-MRI parameters. **(A)** Correlations between the EDSS stage and the TTP **(a–c)**, wash-in rate **(d–f)**, and AUC **(g–i)** of mLVs-SSS in ANMOSD patients. The parameters were correlated with the EDSS stage. **(B)** Receiver operating characteristic (ROC) curve of the TTP **(a–c)**, wash-in rate **(d–f)**, and AUC **(g–i)** of mLVs-SSS in distinguishing ANMOSD from CNMOSD group. **(C)** ROC of the TTP **(a–c)**, wash-in rate **(d–f)**, and AUC **(g–i)** of mLVs-SSS in distinguishing I-ANMOSD group from CNMOSD group.

To investigate whether these parameters were related to the severity of NMOSD, the correlations between the EDSS scale and the parameters (TTP, wash-in rate, or AUC) in ANMOSD were tested, respectively. Our results indicated that TTP values of mLVs-SSS in ANMOSD patients were positively correlated with EDSS scale (**Figures 2Aa–c**). However, wash-in rate values of mLVs-SSS were negatively correlated with the EDSS scale in patients with ANMOSD (**Figures 2Ad–f**). Moreover, AUC values of mLVs-SSS were positively correlated with the EDSS scale in patients with ANMOSD (**Figures 2Ag–i**). Additionally, the logistic regression analysis also showed that the disease severity according to EDSS scale was correlated with the DCE-MRI parameters of mLVs (**Supplementary Table 1**). In a word, DCE-MRI parameters of mLVs-SSS in ANMOSD patients were related to the EDSS scale.

Then, ROC curve analysis was used to evaluate the diagnostic accuracy of DCE-MRI parameters of separate and combined mLVs-SSS for distinguishing ANMOSD from CNMOSD (**Figures 2Ba–i** and **Table 3**). The statistical results showed that TTP values of L-mLVs-SSS, R-mLVs-SSS, L+ R+ Lo-mLVs-SSS, L+R-mLVs-SSS, L + Lo-mLVs-SSS, and R+ Lo-mLVs-SSS had the capacity to distinguished ANMOSD patients from CNMOSD patients with high accuracy, high sensitivity, and specificity (**Figures 2Ba, b** and **Table 3**), while the TTP values of Lo-mLVs-SSS with moderate accuracy, low sensitivity, and high specificity (**Figure 2Bc** and **Table 3**). Wash-in rate values of L-mLVs-SSS, R-mLVs-SSS, Lo-mLVs-SSS, L+ R+ Lo-mLVs-SSS, L+R-mLVs-SSS, L + Lo-mLVs-SSS, and R+ Lo-mLVs-SSS distinguished ANMOSD patients from CNMOSD patients with moderate accuracy, moderate sensitivity, and low specificity (**Figures 2Bd–f** and **Table 3**). Similarly, AUC values of L-mLVs-SSS, R-mLVs-SSS, Lo-mLVs-SSS, L+ R+ Lo-mLVs-SSS, L+R-mLVs-SSS, L + Lo-mLVs-SSS, and R+ Lo-mLVs-SSS distinguished ANMOSD patients from CNMOSD patients with moderate to high accuracy, moderate to low sensitivity, and moderate to low specificity (**Figures 2Bg–i** and **Table 3**).

**Table 3:** Diagnostic accuracy of DCE-MRI parameters of separate and combined mLVs-SSS for distinguishing ANMOSD patients from CNMOSD patients.

	AUROC	95% CI	threshold	Sensitivity (%)	95% CI	Specificity (%)	95% CI
**TTP**							
L-mLVs-SSS	0.9671	0.9228–1.0000	193.0	90.63	74.98–98.02	96.55	82.24–99.91
R-mLVs-SSS	0.9601	0.9132–1.0000	193.0	87.50	71.01–96.49	93.10	77.23–99.15
Lo- mLVs-SSS	0.8847	0.8054–0.9640	125.9	71.88	53.25–86.25	86.21	68.34–96.11
L+R+Lo-mLVs-SSS	0.9564	0.9082–1.0000	469.8	93.75	79.19–99.23	86.21	68.34–96.11
L+R-mLVs-SSS	0.9688	0.9251–1.0000	377.6	93.75	79.19–99.23	93.10	77.23–99.15
L+ Lo-mLVs-SSS	0.9499	0.8982–1.0000	293.7	93.75	79.19–99.23	86.21	68.34–96.11
R+Lo-mLVs-SSS	0.9386	0.8822–1.0000	293.7	87.50	71.01–96.49	86.21	68.34–96.11
**Wash-in rate**							
L-mLVs-SSS	0.7554	0.6289–0.8819	2.429	81.25	63.56–92.79	62.07	42.26–79.31
R-mLVs-SSS	0.6972	0.5564–0.8380	2.282	78.13	60.03–90.72	62.07	42.26–79.31
Lo- mLVs-SSS	0.8190	0.7097–0.9282	2.980	78.13	60.03–90.72	72.41	52.76–87.27
L+R+Lo-mLVs-SSS	0.8039	0.6871–0.9207	7.854	87.50	71.01–96.49	68.97	49.17–84.72
L+R-mLVs-SSS	0.7317	0.5992–0.8642	4.723	84.38	67.21–94.72	62.07	42.26–79.31
L+ Lo-mLVs-SSS	0.8297	0.7216–0.9379	5.092	90.63	74.98–98.02	72.41	52.76–87.27
R+Lo-mLVs-SSS	0.7877	0.6671–0.9083	5.170	81.25	63.56–92.79	72.41	52.76–87.27
**AUC**							
L-mLVs-SSS	0.9030	0.8303–0.9757	53644	84.38	67.21–94.72	79.31	60.28–92.01
R-mLVs-SSS	0.8772	0.7901–0.9642	48790	84.38	67.21–94.72	72.41	52.76–87.27
Lo- mLVs-SSS	0.8836	0.8000–0.9673	23755	78.13	60.03–90.72	82.76	64.23–94.15
L+R+Lo-mLVs-SSS	0.9073	0.8377–0.9770	125130	81.25	63.56–92.79	79.31	60.28–92.01
L+R-mLVs-SSS	0.9073	0.8371–0.9776	103512	81.25	63.56–92.79	79.31	60.28–92.01
L+ Lo-mLVs-SSS	0.9256	0.8637–0.9876	74937	84.38	67.21–94.72	82.76	64.23–94.15
R+Lo-mLVs-SSS	0.8815	0.7990–0.9640	95080	71.88	53.25–86.25	89.66	72.65–97.81

ANMOSD, neuromyelitis optica spectrum disorders patients with acute attack; AUC, area under curve; AUROC, area under receiver operating characteristic curve; CI, confidence interval; CNMOSD, neuromyelitis optica spectrum disorders patients in chronic phase; L-mLVs-SSS, left meningeal lymphatic vessels around superior sagittal sinus; Lo-mLVs-SSS, lower meningeal lymphatic vessels around superior sagittal sinus; R-mLVs-SSS, right meningeal lymphatic vessels around superior sagittal sinus; TTP, time to peak.

Furthermore, ROC curve analysis was used to evaluate the diagnostic accuracy of DCE-MRI parameters of separate and combined mLVs-SSS for distinguishing I-ANMOSD patients from CNMOSD patients (**Figures 2Ca–i** and **Table 4**). Our results showed that TTP values of L-mLVs-SS, R-mLVs-SSS, L+R+Lo-mLVs-SSS, L+R-mLVs-SSS, and L + Lo-mLVs-SSS had the capacity to distinguish I-ANMOSD patients from CNMOSD patients with high accuracy, moderate sensitivity and moderate to high specificity (**Figures 2Ca, b** and **Table 4**), while the TTP values of Lo-mLVs-SSS and R+Lo-mLVs-SSS distinguished I-ANMOSD patients from CNMOSD patients with low to moderate accuracy, low to moderate sensitivity and low specificity (**Figure 2Cc** and **Table 4**). Wash-in rate values of L-mLVs-SSS, R-mLVs-SSS, Lo-mLVs-SSS, L+ R+ Lo-mLVs-SSS, L+R-mLVs-SSS, L + Lo-mLVs-SSS, and R+ Lo-mLVs-SSS distinguished I-ANMOSD patients from CNMOSD patients with low accuracy, low to moderate sensitivity and low specificity (**Figures 2Cd–f** and **Table 4**). AUC values of L-mLVs-SSS, R-mLVs-SSS, Lo-mLVs-SSS, L+ R+ Lo-mLVs-SSS, L+R-mLVs-SSS, L + Lo-mLVs-SSS, and R+ Lo-mLVs-SSS distinguished I-ANMOSD patients from CNMOSD patients with moderate to high accuracy, low to moderate sensitivity, and low specificity (**Figures 2Cg–i**, **Table 4**).

**Table 4:** Diagnostic accuracy of DCE-MRI parameters of separate and combined mLVs-SSS for distinguishing I-ANMOSD patients from CNMOSD patients.

	AUROC	95% CI	threshold	Sensitivity (%)	95% CI	Specificity (%)	95% CI
**TTP**							
L-mLVs-SSS	0.9381	0.8572–1.0000	176.2	88.24	63.56–98.54	89.66	72.65–97.81
R-mLVs-SSS	0.9249	0.8402–1.0000	176.2	82.35	56.57–96.20	86.21	68.34–96.11
Lo- mLVs-SSS	0.7870	0.6581–0.9159	109.1	58.82	32.92–81.56	68.97	49.17–84.72
L+R+Lo-mLVs-SSS	0.9178	0.8315–1.0000	344.0	88.24	63.56–98.54	86.21	68.34–96.11
L+R-mLVs-SSS	0.9412	0.8613–1.0000	377.6	88.24	63.56–98.54	93.10	77.23–99.15
L+ Lo-mLVs-SSS	0.9057	0.8137–0.9977	293.7	88.24	63.56–98.54	86.21	68.34–96.11
R+Lo-mLVs-SSS	0.8844	0.7856–0.9831	276.9	82.35	56.57–96.20	79.31	60.28–92.01
**Wash in rate**							
L-mLVs-SSS	0.6450	0.4838–0.8063	2.429	70.59	44.04–89.69	62.07	42.26–79.31
R-mLVs-SSS	0.5963	0.4308–0.7619	2.282	64.71	38.33–85.79	62.07	42.26–79.31
Lo- mLVs-SSS	0.7424	0.5994–0.8854	2.759	70.59	44.04–89.69	68.97	49.17–84.72
L+R+Lo-mLVs-SSS	0.7262	0.5759–0.8765	7.584	82.35	56.57–96.20	68.97	49.17–84.72
L+R-mLVs-SSS	0.6268	0.4648–0.7887	4.723	76.47	50.10–93.19	62.07	42.26–79.31
L+ Lo-mLVs-SSS	0.7525	0.6068– 0.8983	5.092	88.24	63.56–98.54	72.41	52.76–87.27
R+Lo-mLVs-SSS	0.7079	0.5549–0.8609	5.170	70.59	44.04–89.69	72.41	52.76–87.27
**AUC**							
L-mLVs-SSS	0.9030	0.8303–0.9757	53644	70.59	44.04–89.69	79.31	60.28–92.01
R-mLVs-SSS	0.8093	0.6764–0.9422	47518	82.35	56.57–96.20	68.97	49.17–84.72
Lo- mLVs-SSS	0.8093	0.6793–0.9394	19853	82.35	56.57–96.20	65.52	45.67–82.06
L+R+Lo-mLVs-SSS	0.8458	0.7351–0.9566	125130	70.59	44.04–89.69	79.31	60.28–92.01
L+R-mLVs-SSS	0.8458	0.7336–0.9581	92146	82.35	56.57–96.20	68.97	49.17–84.72
L+ Lo-mLVs-SSS	0.8682	0.7636–0.9727	61531	88.24	63.56–98.54	68.97	49.17–84.72
R+Lo-mLVs-SSS	0.8134	0.6873–0.9395	62213	88.24	63.56–98.54	62.07	42.26–79.31

I-ANMOSD, neuromyelitis optica spectrum disorders patients with acute attack (EDSS ≤ 4.5); AUC, area under curve; AUROC, area under receiver operating characteristic curve; CI, confidence interval; CNMOSD, neuromyelitis optica spectrum disorders patients in chronic phase; L-mLVs-SSS, left meningeal lymphatic vessels around superior sagittal sinus; Lo-mLVs-SSS, lower meningeal lymphatic vessels around superior sagittal sinus; R-mLVs-SSS, right meningeal lymphatic vessels around superior sagittal sinus; TTP, time to peak.

In summary, our results showed that DCE-MRI parameters of L-mLVs-SSS, R-mLVs-SSS, L+ R+ Lo-mLVs-SSS, L+R-mLVs-SSS, L + Lo-mLVs-SSS, and R+ Lo-mLVs-SSS could distinguish ANMOSD or I-ANMOSD from CNMOSD, especially the TTP values of L-mLVs-SSS, R-mLVs-SSS, and L+R-mLVs-SSS, they distinguished ANMOSD or I-ANMOSD from CNMOSD patients with high sensitivity and specificity.

### Measurement of Cross-Sectional Areas of mLVs-SSS

**Figure 3:**
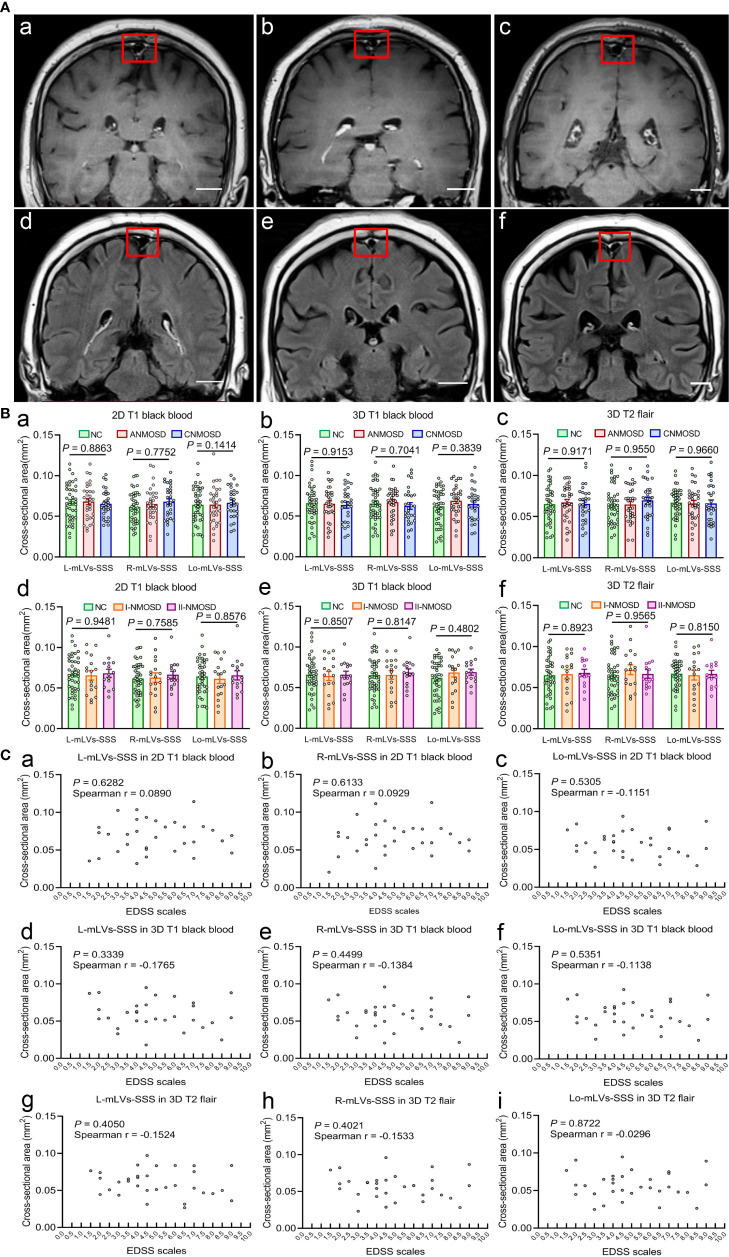
Visualization and measurement of mLVs-SSS in different groups by high-solution MRI sequences. **(A)** Visualization of mLVs-SSS in NC **(a, d)**, ANMOSD **(b, e)**, and CNMOSD **(c, f)** by 2D T1 black-blood **(a–c)** and 3D T2 flair **(d–f)** sequence. The red rectangles stand for the three mLVs-SSS (L-mLVs-SSS, R-mLVs-SSS, Lo-mLVs-SSS), L-mLVs-SSS, R-mLVs-SSS and Lo- mLVs-SSS represents the left, right and lower mLVs-SSS, respectively. Scale bar, 2 cm. **(B)** Measurement and comparison of the cross-sectional area of mLVs-SSS in different MRI sequences. The cross-sectional area of mLVs-SSS in three groups (NC, ANMOSD, CNMOSD) were not significantly different with each group in 2D T1 black-blood **(a)**, 3D T1 black-blood **(b)** and 3D T2 flair **(c)** sequences. The cross-sectional area of mLVs-SSS in NC and ANMOSD patients (I-ANMOSD group, II-ANMOSD group) in 2D T1 black-blood **(d)**, 3D T1 black-blood **(e)** and 3D T2 flair **(f)** sequences. There was no difference between groups in all MRI sequences. I-ANMOSD group, EDSS stage ≤ 4.5; II-ANMOSD group, EDSS stage > 4.5. **(C)** Correlations between the EDSS scale and the cross-sectional area of mLVs-SSS in ANMOSD patients in 2D T1 black-blood **(a–c)**, 3D T1 black-blood **(d–f)** and 3D T2 flair **(g–i)** sequences. The cross-sectional area of mLVs-SSS was not correlated with the EDSS scale.

After DCE-MRI, in order to measure the cross-sectional areas of the mLVs-SSS, every participant completed three high-resolution MRI scans continuously thirty minutes after injection of gadobutrol, including 2D T1 black-blood, 3D T1 black-blood, and 3D T2 flair MRI scans. The cross-sectional areas of mLVs-SSS (L-, R-, and Lo-mLVs-SSS) were measured in each participant of the three MRI sequences, respectively. The representative mLVs-SSS in 2D T1 black-blood (**Figures 3Aa–c**) and 3D T2 flair (**Figures 3Ad–f**) images in NC, ANMOSD, and CNMOSD patients were shown in **Figure 3A**. The results showed that no significant differences in the largest cross-sectional areas of the mLVs-SSS among NC, ANMOSD, and CNMOSD in 2D T1 black-blood (L-mLVs-SSS: F = 0.1208, *P* = 0.8863; R-mLVs-SSS: F = 0.7752, *P* = 0.4634; Lo-mLVs-SSS: F = 0.1414, *P* = 0.8683. **Figure 3Ba**), 3D T1 black-blood (L-mLVs-SSS: F = 0.0886, *P* = 0.9153; R-mLVs-SSS: F = 0.3521, *P* = 0.7041; Lo-mLVs-SSS: F = 0.9536, *P* = 0.3889; **Figure 3Bb**), and 3D T2 flair (L-mLVs-SSS: F = 0.0866, *P* = 0.9171; R-mLVs-SSS: F = 0.0514, *P* = 0.9500; Lo-mLVs-SSS: F = 0.0346, *P* = 0.9660; **Figure 3Bc**), respectively.

Then, the largest cross-sectional areas of the mLVs-SSS in I-ANMOSD and II- ANMOSD groups were compared with that in NC in different MRI sequences. Our results showed that there were no differences in the largest cross-sectional areas of the mLVs-SSS in NC, I-ANMOSD, and II-ANMOSD in 2D T1 black-blood (L-mLVs-SSS: F = 0.0533, *P* = 0.9481; R-mLVs-SSS: F = 0.2775, *P* = 0.7585; Lo-mLVs-SSS: F = 0.1540, *P* = 0.8576; **Figure 3Bd**), 3D T1 black-blood (L-mLVs-SSS: F = 0.1620, *P* = 0.8507; R-mLVs-SSS: F = 0.2056, *P* = 0.8147; Lo-mLVs-SSS: F = 0.7412, *P* = 0.4802; **Figure 3Be**) and 3D T2 flair (L-mLVs-SSS: F = 0.1141, *P* = 0.8923; R-mLVs-SSS: F = 0.0446, *P* = 0.9565; Lo-mLVs-SSS: F = 0.2052, *P* = 0.8150; **Figure 3Bf**), respectively.

Lastly, the correlation between the EDSS scale and the largest cross-sectional areas of mLVs-SSS (L, R, and Lo-mLVs-SSS) in ANMOSD patients were tested in different MRI sequences. The results indicated that the largest cross-sectional areas of mLVs-SSS in ANMOSD were not significantly correlated with EDSS scale in 2D T1 black-blood (**Figures 3Ca–c**), 3D T1 black-blood (**Figures 3Cd–f**), and 3D T2 flair (**Figures 3Cg–i**), respectively.

Additionally, we further compared the average and minimal cross-sectional areas of the mLVs-SSS in different groups separately. The results showed that the average and minimal cross-sectional areas of the mLVs-SSS were not significantly different among NC, ANMOSD, and CNMOSD patients in 2D T1 black-blood, 3D T1 black-blood and 3D T2 flair sequences, respectively. The F and *P* values were shown in **Supplementary Table 2**. Furthermore, no significant differences were found in the average and minimal cross-sectional areas of mLVs-SSS among NC, I-ANMOSD and II-ANMOSD in 2D T1 black-blood, 3D T1 black-blood, and 3D T2 flair sequences, respectively. The detailed F and *P* values were shown in **Supplementary Table 3**. The average and minimal cross-sectional areas of mLVs-SSS in ANMOSD were not significantly correlated with EDSS scale in 2D T1 black-blood, 3D T1 black-blood, and 3D T2 flair sequences, respectively. The r and *P* values were shown in **Supplementary Table 4**.

In summary, our results showed that the cross-sectional area of mLVs-SSS in NMOSD (ANMOSD and CNMOSD) were not significantly different from that in NC, and the cross-sectional area of mLVs-SSS in ANMOSD patients was not associated with EDSS scale.

## Discussion

In this study, DCE-MRI was used to quantitatively assess the mLVs-SSS flow in NC and NMOSD patients. The slow lymphatic flow was found in ANMOSD patients instead of CNMOSD patients and NC. The correlation analysis showed that DCE-MRI parameters of mLVs-SSS in ANMOSD patients were correlated with the disease severity. Then, the cross-sectional areas of the mLVs-SSS in ANMOSD patients, CNMOSD patients and NC in different high-resolution MRI sequences were measured, and no significant difference of the size of mLVs-SSS was found. Our findings demonstrated that meningeal lymphatic flow impaired in ANMOSD patients rather than that in CNMOSD patients.

The conventional contrast enhanced MRI was used to assess the function of glymphatic system after intrathecal injection of gadolinium at different time points, which was invasive, discontinuous, and unacceptable (24). In our study, DCE-MRI was performed to acquire head MRI images successively after intravenous gadolinium injection, and it was accurate, convenient, and acceptable. The signal intensity of mLVs-SSS at different time points were analyzed and exhibited by the TIC, which reflects the flow of mLVs-SSS. Our study indicated that the meningeal lymphatic flow around SSS is significantly impaired in ANMOSD patients compared to NC and CNMOSD patients. Additionally, the slower mLVs flow was correlated with the severity of ANMOSD evaluated by EDSS scale.

Numerous studies have demonstrated that mLVs played an important role in maintaining brain tissue homeostasis and dysfunction of mLVs was involved in the progression of neurodegenerative diseases (6, 9, 27). Dysfunction of mLVs would aggravate deposition of Aβ in brain tissue and cognitive impairment in AD mouse model (28–30). However, mLVs also drain some immune cells, antigens, antibodies and lymphocytes to CLNs (6, 10, 31), suggesting that mLVs may be involved in the regulation of neuroimmune. Previous studies have suggested that mLVs were physiologically or pathologically altered in neuroinflammatory disease, such as multiple sclerosis (MS) (32). In MS patients and some animal models, changes in tertiary lymphoid structures also occurred (33, 34). Our findings indicated that meningeal lymphatic function was impaired in ANMOSD patients, and it also indicated that mLVs’ dysfunction was associated with the neuroinflammatory disease.

It is not clear about the specific mechanism leading to the mLVs drainage dysfunction in neuroinflammatory disease, we inferred that it might be caused by the acute inflammation in the meanings. The meninges around the brain are filled with various types of immune cells that not only provide immune surveillance but also influence brain function (32). When CNS inflammation occurs, immune cells in the meninges participate in the inflammatory response, which in turn produces a series of inflammatory factors, such as IL-1, TNF, CCL-2, and IL-17A (35). These inflammatory factors may damage the meningeal lymphatic endothelial cells, destroy the tight connection of lymphatic vascular endothelial cells, and affect the drainage function of lymphatic vessels (35, 36). The mLVs worked as an important drainage pathway for immune cells’ excretion from CNS to the periphery lymph nodes, and its drainage dysfunction will lead to the accumulation of antigens, antibodies and immune cells in CNS. The impaired AQP4–IgG excretion and autoimmune inflammation might interact as both cause and effect, which ultimately leads to immune damage of CNS. The increase of AQP4-IgG would activate complement, and the depositional of complement would activate the terminal complement pathway, which might lead to the formation of the membrane attack complex (MAC) (37, 38) and the antibody-dependent cell-mediated cytotoxicity, which involved in the inflammatory demyelination (39). Moreover, the bound of astrocyte with Fc region of the AQP4-IgG would also activate several infiltrating immune cells, such as macrophages and neutrophils (40, 41). All of the above pathological processes finally caused demyelination and necrosis in the certain CNS regions that express high levels of AQP4, such as optic nerve and spinal cord. Moreover, the mLVs played an important role in connecting the central and peripheral immune system (11), and some AQP4–IgG in peripheral blood of NMOSD patients would enter CNS through the destroyed blood brain barrier (BBB). As shown in the previous studies about neuroinflammatory disease, BBB has been destroyed by inflammatory factors, such as IL-1, TNF-, CCL-2, and IL-17A, through degrading some tight-junction proteins (35). The dysfunction of mLVs would reduce the excretion of inflammatory factors from CSF, and aggravate the BBB damage further. Damage to the BBB, in turn, stimulates AQP4–IgG in the peripheral blood to enter the CNS.

Our study showed that dysfunction of mLVs occurred in NMOSD patients with acute attack, and the dysfunction was associated with the disease severity. However, the function of mLVs was almost normal in NMOSD patients with chronic phase. This maybe because the meningeal inflammation is more severe in acute phase of NMOSD, which leads to acute impairment of lymphatic function, while in chronic phase of NMOSD, the meningeal inflammation is less severe and lymphatic drainage function is relatively normal. Moreover, we found that the drainage dysfunction of mLVs has occurred in patients with mild neurological impairment of ANMOSD (I-ANMOSD), which further suggested that dysfunction of mLVs might occur in the early onset of the disease. Meanwhile, the dysfunction of mLVs might involve in the disease progression. Therefore, promoting or restoring the drainage function of mLVs might be a new target for the treatment of NMOSD acute attack. Such as treatment with the recombinant of vascular endothelial growth factor C (VEGF-C) or the adeno-associated virus serotype 1 (AAV1) vector expressing VEGF-C. Previous research has demonstrated that treatment with recombinant VEGF-C increases the diameter of meningeal lymphatic vessels (6). Additionally, delivery of VEGF-C by adenoviral gene therapy could efficiently promote and boost peripheral lymphatic sprouting and function (42, 43). However, AQP-4 is the predominant water channel expressed in perivascular astrocytic end-feet and astrocyte membranes that face the pia mater and ependymal cells, and it played an important role in the glymphatic clearance, so it is likely that the glymphatic system impairment is also involved in the pathology of NMOSD. Nevertheless, whether glymphatic system dysfunction occurred in NMOSD patients was still unknown and need to be evaluated further.

DCE-MRI might provide a novel method to predict the acute relapse of NMOSD. Up to now, the NMOSD diagnosis criteria mainly depends on the detection of AQP4-IgG, clinical characteristics and MRI images. Nevertheless, the sensitivity of traditional detection methods is limited and it always takes a long time to get the results. The clinical symptoms, such as paresthesia, are often lack of objective criteria for evaluation. Through evaluating the function of mLVs, DCE-MRI will provide objective evidence to predict the relapse of NMOSD. The exact diagnosis is of great significance for the timely treatment and prevention of disease progression, as patients with NMOSD relapses require timely immunomodulatory therapy, including high-dose steroids (44, 45), IV immunoglobulin (IVIg) therapy (45, 46) and plasma exchange (44, 47, 48) in the acute phase. In this study, the DCE-MRI parameters, especially the TTP values of L-mLVs-SSS, R-mLVs-SSS, and L+R-mLVs-SSS, had the capacity to distinguished ANMOSD patients from CNMOSD patients with high accuracy, high sensitivity and specificity.

A recent report suggested that the autoimmune response might link with lymphedema. However, our findings demonstrated that the size of mLVs did not change, as the cross-sectional area of mLVs in NMOSD were not different with that in NC. This suggested that lymphedema or dilation of mLVs were not main culprit in dysfunction of mLVs.

In summary, our study demonstrates that the impairment of meningeal lymphatic flow is associated with the relapse and deterioration of the NMOSD. Greater understanding of meningeal lymphatic flow dysfunction in NMOSD will provide better insights into the pathogenesis of NMOSD. Improvement of the meningeal lymphatic function might be meaningful for the diagnosis and treatment of NMOSD, and even slow the progression of the disease.

## Data Availability Statement

The original contributions presented in the study are included in the article/**Supplementary Material**. Further inquiries can be directed to the corresponding authors.

## Ethics Statement

The studies involving human participants were reviewed and approved by the Institutional Ethics Committees of The First Affiliated Hospital of Zhengzhou University. The patients/participants provided their written informed consent to participate in this study. Written informed consent was obtained from the individual(s) for the publication of any potentially identifiable images or data included in this article.

## Author Contributions

XJW conceived and designed the experiments. XJW coordinated the whole project. XD, XXW, DL, YF, and YPZ were responsible for the initial assessment and diagnosing patients. XD, XXW, HT, and YF were responsible for assessing, documenting their patients’ health information. HT, SX, CQ, and LM performed the image analysis. XXW, HL, QZ, and XD provided statistical analysis and technical support. XJW, XD, XXW, HL, and DL participated in final data analysis and interpretation. XJW, XXW, HT, and XD did most of the writing with input from other authors. All authors contributed to the article and approved the submitted version.

## Funding

XJW was supported by grants from the National Natural Science Foundation of China (no. 81873791, 81471307) and the Natural Science Foundation of Henan Province for Excellent Young Scholars (no. 202300410357).

## Conflict of Interest

The authors declare that the research was conducted in the absence of any commercial or financial relationships that could be construed as a potential conflict of interest.
