# Supplementary material for: Impaired Meningeal Lymphatic Flow in NMOSD Patients With Acute Attack
Source: Front Immunol. 2021 Jun 14;12:692051. doi: 10.3389/fimmu.2021.692051 (PMC8236891; doi:10.3389/fimmu.2021.692051)
Supplement: Supplementary file 1 [file Table_1.docx]

**Supplementary Table 1 Logistic regression of the clinical characteristics and DCE-MRI parameters in ANMOSD patients**

|  |  |  |  | Number  of attacks | MRI lesion | | | |  |
| --- | --- | --- | --- | --- | --- | --- | --- | --- | --- |
|  | Sex | age | Duration |  | Spinal cord | Optic nerve | Brain | others | EDSS |
| **TTP** |  |  |  |  |  |  |  |  |  |
| L-mLVs-SSS | 0.294 | 0.247 | 0.295 | 0.171 | 0.214 | 0.159 | 0.968 | 0.316 | 0.031^*^ |
| R-mLVs-SSS | 0.217 | 0.591 | 0.197 | 0.108 | 0.523 | 0.581 | 0.673 | 0.146 | 0.024^*^ |
| Lo-mLVs-SSS | 0.901 | 0.961 | 0.137 | 0.964 | 0.554 | 0.872 | 0.203 | 0.342 | 0.009^*^ |
| **Wash in rate** |  |  |  |  |  |  |  |  |  |
| L-mLVs-SSS | 0.326 | 0.775 | 0.234 | 0.590 | 0.568 | 0.250 | 0.243 | 0.817 | 0.036^*^ |
| R-mLVs-SSS | 0.459 | 0.077 | 0.314 | 0.100 | 0.118 | 0.740 | 0.099 | 0.129 | 0.030^*^ |
| Lo-mLVs-SSS | 0.399 | 0.655 | 0.365 | 0.272 | 0.631 | 0.706 | 0.290 | 0.320 | 0.019^*^ |
| **AUC** |  |  |  |  |  |  |  |  |  |
| L-mLVs-SSS | 0.414 | 0.548 | 0.272 | 0.726 | 0.192 | 0.069 | 0.089 | 0.655 | 0.031^*^ |
| R-mLVs-SSS | 0.472 | 0.432 | 0.363 | 0.803 | 0.819 | 0.430 | 0.646 | 0.594 | 0.043^*^ |
| Lo-mLVs-SSS | 0.171 | 0.643 | 0.372 | 0.348 | 0.254 | 0.135 | 0.058 | 0.877 | 0.019^*^ |

Abbreviations: ANMOSD = Neuromyelitis optica spectrum disorders patients with acute attack; AUC = area under curve; DCE-MRI = dynamic contrast-enhanced magnetic resonance imaging; EDSS = Expanded Disability Status Scale; L-mLVs-SSS = left meningeal lymphatic vessels around superior sagittal sinus; Lo-mLVs-SSS = lower meningeal lymphatic vessels around superior sagittal sinus; R-mLVs-SSS = right meningeal lymphatic vessels around superior sagittal sinus; TTP = time to peak.^*^*P* < 0.05.
