# Supplementary material for: Impaired Meningeal Lymphatic Flow in NMOSD Patients With Acute Attack
Source: Front Immunol. 2021 Jun 14;12:692051. doi: 10.3389/fimmu.2021.692051 (PMC8236891; doi:10.3389/fimmu.2021.692051)
Supplement: Supplementary file 2 [file Table_2.docx]

**Supplementary Table 2 Statistical analysis of the** **cross-sectional area of mLVs-SSS among NC, ANMOSD and CNMOSD groups in different MRI sequences**

|  | **The average cross-sectional areas** | | **The minimal cross-sectional areas** | |
| --- | --- | --- | --- | --- |
|  | F | *P* | F | *P* |
| **2D T1 black-blood** |  |  |  |  |
| L-mLVs-SSS | 0.0782 | 0.9248 | 0.1569 | 0.8550 |
| R-mLVs-SSS | 0.3139 | 0.7313 | 0.5142 | 0.5996 |
| Lo-mLVs-SSS | 0.0857 | 0.9180 | 0.1273 | 0.8806 |
| **3D T1 black-blood** |  |  |  |  |
| L-mLVs-SSS | 0.1764 | 0.8385 | 0.1365 | 0.8726 |
| R-mLVs-SSS | 0.5501 | 0.5786 | 0.6212 | 0.5394 |
| Lo-mLVs-SSS | 0.4711 | 0.6257 | 0.6680 | 0.5151 |
| **3D T2 Flair** |  |  |  |  |
| L-mLVs-SSS | 0.1758 | 0.8391 | 0.2152 | 0.8068 |
| R-mLVs-SSS | 0.1763 | 0.8361 | 0.1120 | 0.8942 |
| Lo-mLVs-SSS | 0.0251 | 0.0972 | 0.2164 | 0.8058 |

Abbreviations: ANMOSD = neuromyelitis optica spectrum disorders patients with acute attack; CNMOSD = neuromyelitis optica spectrum disorders patients in chronic phase; L-mLVs-SSS = left meningeal lymphatic vessels around superior sagittal sinus; Lo-mLVs-SSS = lower meningeal lymphatic vessels around superior sagittal sinus; NC = normal controls; R-mLVs-SSS = right meningeal lymphatic vessels around superior sagittal sinus.
