# Supplementary material for: Impaired Meningeal Lymphatic Flow in NMOSD Patients With Acute Attack
Source: Front Immunol. 2021 Jun 14;12:692051. doi: 10.3389/fimmu.2021.692051 (PMC8236891; doi:10.3389/fimmu.2021.692051)
Supplement: Supplementary file 3 [file Table_3.docx]

**Supplementary Table 3 Statistical analysis of the cross-sectional area of mLVs-SSS among NC, Ⅰ-ANMOSD and Ⅱ-ANMOSD groups in different MRI sequences**

|  | **The average cross-sectional areas** | | **The minimal cross-sectional areas** | |
| --- | --- | --- | --- | --- |
|  | F | *P* | F | *P* |
| **2D T1 black-blood** |  |  |  |  |
| L-mLVs-SSS | 0.2282 | 0.7966 | 0.3350 | 0.7165 |
| R-mLVs-SSS | 0.2422 | 0.7855 | 0.3626 | 0.6972 |
| Lo-mLVs-SSS | 0.3975 | 0.6735 | 0.3711 | 0.6913 |
| **3D T1 black-blood** |  |  |  |  |
| L-mLVs-SSS | 0.2651 | 0.7679 | 0.2577 | 0.7736 |
| R-mLVs-SSS | 0.3285 | 0.7211 | 0.1973 | 0.8214 |
| Lo-mLVs-SSS | 0.1705 | 0.8436 | 0.6309 | 0.5351 |
| **3D T2 Flair** |  |  |  |  |
| L-mLVs-SSS | 0.2694 | 0.7646 | 0.3141 | 0.7315 |
| R-mLVs-SSS | 0.1930 | 0.8249 | 0.2778 | 0.7583 |
| Lo-mLVs-SSS | 0.3096 | 0.7438 | 0.3720 | 0.6907 |

Abbreviations: Ⅰ-ANMOSD = neuromyelitis optica spectrum disorders patients with acute attack (EDSS ≤ 4.5); Ⅱ-ANMOSD = neuromyelitis optica spectrum disorders patients with acute attack (EDSS > 4.5); L-mLVs-SSS = left meningeal lymphatic vessels around superior sagittal sinus; Lo-mLVs-SSS = lower meningeal lymphatic vessels around superior sagittal sinus; NC = normal controls; R-mLVs-SSS = right meningeal lymphatic vessels around superior sagittal sinus.
