# Supplementary material for: Impaired Meningeal Lymphatic Flow in NMOSD Patients With Acute Attack
Source: Front Immunol. 2021 Jun 14;12:692051. doi: 10.3389/fimmu.2021.692051 (PMC8236891; doi:10.3389/fimmu.2021.692051)
Supplement: Supplementary file 4 [file Table_4.docx]

**Supplementary Table 4 Spearman correlation analysis of the EDSS scales and the cross-sectional area of mLVs-SSS in ANMOSD groups in different MRI sequences**

|  | **The average cross-sectional areas** | | **The minimal cross-sectional areas** | |
| --- | --- | --- | --- | --- |
|  | r | *P* | r | *P* |
| **2D T1 black-blood** |  |  |  |  |
| L-mLVs-SSS | 0.0837 | 0.6497 | 0.0577 | 0.7536 |
| R-mLVs-SSS | 0.1215 | 0.5076 | 0.1004 | 0.5846 |
| Lo-mLVs-SSS | 0.0883 | 0.6310 | 0.1175 | 0.5219 |
| **3D T1 black-blood** |  |  |  |  |
| L-mLVs-SSS | 0.1009 | 0.5825 | 0.1202 | 0.5121 |
| R-mLVs-SSS | 0.0967 | 0.5985 | 0.0842 | 0.6468 |
| Lo-mLVs-SSS | 0.0769 | 0.6759 | 0.0123 | 0.9467 |
| **3D T2 Flair** |  |  |  |  |
| L-mLVs-SSS | 0.0910 | 0.6203 | 0.1179 | 0.5206 |
| R-mLVs-SSS | 0.0905 | 0.6225 | 0.0949 | 0.6055 |
| Lo-mLVs-SSS | 0.1031 | 0.5743 | 0.0831 | 0.6511 |

Abbreviations: ANMOSD = neuromyelitis optica spectrum disorders patients with acute attack; EDSS = Expanded Disability Status Scale; L-mLVs-SSS = left meningeal lymphatic vessels around superior sagittal sinus; Lo-mLVs-SSS = lower meningeal lymphatic vessels around superior sagittal sinus; NC = normal controls; R-mLVs-SSS = right meningeal lymphatic vessels around superior sagittal sinus.
